# Hemodynamic effects of acute hyperoxia: systematic review and meta-analysis

**DOI:** 10.1186/s13054-018-1968-2

**Published:** 2018-02-25

**Authors:** Bob Smit, Yvo M. Smulders, Johannes C. van der Wouden, Heleen M. Oudemans-van Straaten, Angelique M. E. Spoelstra-de Man

**Affiliations:** 1 Department of Intensive Care, VU University Medical Center, De Boelelaan 1117, 1007 MB Amsterdam, the Netherlands; 2 Department of Internal Medicine, VU University Medical Center, Amsterdam, the Netherlands; 3 Department of General Practice and Elderly Care Medicine, Amsterdam Public Health research institute, VU University Medical Center, Amsterdam, the Netherlands

**Keywords:** Hyperoxia, Oxygen, Hemodynamics, Systematic review, Meta-analysis

## Abstract

**Background:**

In clinical practice, oxygen is generally administered to patients with the intention of increasing oxygen delivery. Supplemental oxygen may, however, cause arterial hyperoxia, which is associated with hemodynamic alterations. We performed a systematic review and meta-analysis of the literature to determine the effect of hyperoxia on central hemodynamics and oxygen delivery in healthy volunteers and cardiovascular-compromised patients.

**Methods:**

PubMed and EMBASE were searched up to March 2017. Studies with adult humans investigating changes in central hemodynamics or oxygen delivery induced by acute normobaric hyperoxia were included. Studies focusing on lung, retinal, or brain parameters were not included. We extracted subject and oxygen exposure characteristics, indexed and unindexed values for heart rate, stroke volume, cardiac output, mean arterial pressure (MAP), systemic vascular resistance, and oxygen delivery during normoxia and hyperoxia. For quantitative synthesis of the data, a random-effects ratio of means (RoM) model was used.

**Results:**

We identified 33 studies with 42 datasets. Study categories included healthy volunteers (*n* = 22 datasets), patients with coronary artery disease (CAD; *n* = 6), heart failure (HF; *n* = 6), coronary artery bypass graft (CABG; *n* = 3) and sepsis (*n* = 5). Hyperoxia (arterial oxygen tension of 234–617 mmHg) reduced cardiac output (CO) by 10–15% in both healthy volunteers (−10.2%, 95% confidence interval (CI) −12.9% to −7.3%) and CAD (−9.6%, 95% CI −12.3% to −6.9%) or HF patients (−15.2%, 95% CI −21.7% to −8.2%). No significant changes in cardiac output were seen in CABG or septic patients (−3%). Systemic vascular resistance increased remarkably in patients with heart failure (24.6%, 95% CI 19.3% to 30.1%). In healthy volunteers, and those with CAD and CABG, the effect was smaller (11–16%) and was virtually absent in patients with sepsis (4.3%, 95% CI −3.2% to 12.3%). No notable effect on MAP was found in any group (2–3%). Oxygen delivery was not altered by hyperoxia. Considerable heterogeneity existed between study results, likely due to methodological differences.

**Conclusions:**

Hyperoxia may considerably decrease cardiac output and increase systemic vascular resistance, but effects differ between patient categories. Heart failure patients were the most sensitive while no hemodynamic effects were seen in septic patients. There is currently no evidence supporting the notion that oxygen supplementation increases oxygen delivery.

**Electronic supplementary material:**

The online version of this article (10.1186/s13054-018-1968-2) contains supplementary material, which is available to authorized users.

## Background

In critical care and emergency medicine, oxygen is frequently administered to ensure satisfactory oxygen delivery to organs. To correct or prevent hypoxia, oxygen is often supplemented superfluously which may lead to hyperoxia (a higher than normal arterial partial pressure of oxygen (P_a_O_2_)).

Both negative and positive clinical consequences are ascribed to hyperoxia. It is associated with increased intensive care unit (ICU) mortality [1–3] and increased myocardial [4] and cerebral infarction size [5]. However, hyperoxia has also been associated with beneficial effects such as improved organ function after cardiac arrest [6] and, in animal models, hyperoxia has been shown to induce a redistribution of blood flow to vital organs [7, 8].

The cardiovascular effects of oxygen could play an important role in the aforementioned clinical outcomes. Reported hemodynamic effects include peripheral vasoconstriction and reduced cardiac output (CO) [9]. These effects may exacerbate pre-existing perfusion disturbances and, therefore, reduce tissue oxygen delivery [10]. On the other hand, hyperoxic peripheral vasoconstriction may improve circulatory shock, potentially reducing the need for fluid and vasopressor resuscitation [11, 12].

The magnitude of hyperoxia-induced hemodynamic alterations is currently unclear, as is the generalizability of the effects to different types of patients. In this systematic review and meta-analysis, we aim to provide an overview of the evidence of changes in hemodynamics and oxygen delivery induced by oxygen supplementation in healthy volunteers and patients with cardiovascular disease or sepsis.

## Methods

### Search strategy

We searched PubMed and EMBASE for eligible studies published up to March 2017. The search query consisted of various keywords related to the domains of hemodynamics, hyperoxia, and humans (Additional file 1). These separate domains were combined with the AND operator. References of included studies were screened for publications that were not identified in the search.

### Study selection

Studies were screened in three separate phases. Phase 1 consisted of screening based on title by one of the authors (BS); obviously irrelevant articles were excluded. During phase 2, two authors (BS, AMESdM) selected articles based on the abstract for full text screening in phase 3. Inclusion criteria were studies with adults that investigated the effect of hyperoxia induced by short-term (< 6 h) inhalation of oxygen on systemic hemodynamic parameters (heart rate (HR), mean arterial pressure (MAP), CO, stroke volume (SV), systemic vascular resistance (SVR), and oxygen delivery (DO_2_)) in comparison with normoxia. For studies with healthy volunteers, normoxia was defined as a fraction of inspired oxygen (F_I_O_2_) of 21%. For studies with patients, a higher baseline F_I_O_2_ was accepted before hyperoxia induction. Studies had to report on a combination of heart rate and stroke volume or cardiac output, or on oxygen delivery. We excluded studies involving hyper- or hypobaria, chronic lung disease, sleep disorders (e.g., apnea studies), resuscitation (e.g., use of oxygen after cardiac arrest), pregnancy or childbirth, and changes in the inspired gas fraction other than oxygen and long-term hyperoxia (> 6 h). Studies on the effect of hyperoxia during exercise were included only if they contained data during rest. We did not include studies measuring solely lung, retinal, or brain parameters.

### Data extraction

We extracted the following data from each study: the first author’s last name, publication year, number of subjects, method of oxygen administration, main measurement method for cardiac indices, F_I_O_2_, P_a_O_2_, and exposure time. For the parameters of interest (HR, SV, CO, MAP, SVR, DO_2_) we extracted the mean and standard deviation (SD) or standard error of the mean (SEM) during normoxia and hyperoxia, correlation coefficients, and change from baseline along with SD/SEM where available. Both indexed and unindexed parameters (e.g., cardiac output and cardiac index) were extracted. If sufficient patient-level data were reported, missing parameters were calculated with conventional formulae [13]. If a study investigated multiple oxygen dosages, we included only the highest dose in this analysis. If measurements were made at multiple timepoints, we extracted the data from the timepoint that was closest to that of the mean of other studies.

### Risk of bias

For the assessment of the risk of bias of the included studies, we used a modified version of a quality assessment tool for pre-post studies without a control group (see Additional file 2) [14]. The tool consists of 11 questions which pertain to the presence of an adequate description of the study objective, study population, sample size calculation, intervention and its verification, the application of randomization, the stability of the study subjects, possible carry-over effects, participant blinding, assessor blinding, and, finally, statistics and statistical tests. Possible answers were “yes” (low risk of bias), “no” (high risk of bias), “uncertain” (uncertain risk of bias) and “not applicable”.

### Data synthesis and analysis

Effect sizes of the individual studies are presented as the ratio of means (RoM) with 95% confidence intervals (CIs) [15], adjusted for correlated measurements (see Additional file 3), and were pooled by a random effects model [16]. For ease of interpretation, we converted RoM to percentage change (%) using the following formula: (RoM – 1) × 100. Due to the obvious health differences between volunteers and patients, studies with either population were analyzed separately. Heterogeneity was assessed by the *I*^2^ statistic and is reported along with its 95% CI [17]. For studies that did not report correlation coefficients between pre- and post-test measurements, we imputed the average correlation coefficient from other studies. A sensitivity analysis was performed to test the influence of the imputed coefficients. The likelihood of publication bias was assessed by visual inspection of the funnel plot for studies measuring CO. All calculations were made with Microsoft Excel [18]. Graphs were made using Graphad Prism 7.0 (GraphPad Software, Inc., La Jolla, USA).

## Results

### Search results and study characteristics

**Fig. 1:**
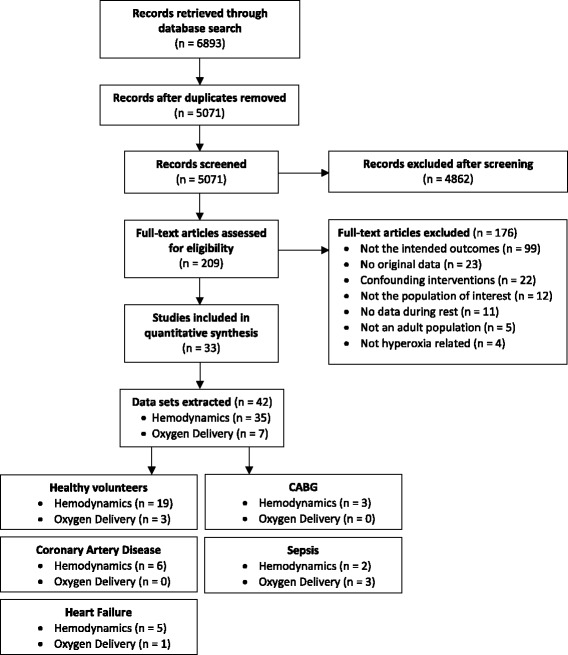
Flow diagram of the included and excluded studies. The flow chart for the inclusion and exclusion of studies for the current meta-analysis. *CABG* coronary artery bypass graft

We found 6893 articles in the online databases of PubMed and EMBASE (Fig. 1). After screening and eligibility assessment, a total of 33 studies were included for this meta-analysis, reporting measurements in healthy volunteers (*n* = 19 studies), patients with coronary artery disease (CAD; *n* = 6), heart failure (HF; *n* = 5), post-coronary artery bypass graft (CABG) surgery (*n* = 3), or sepsis (*n* = 3). From these studies, 42 datasets could be extracted (see Table 1 for an overview of the study characteristics from studies with healthy volunteers, and Table 2 for studies with patients). Other patient populations found, but excluded from this analysis, were patients with pulmonary arterial hypertension and cirrhosis. The included studies were published between 1958 and 2017, with the majority (55%) being published after 2000. The study population sizes were relatively small, ranging between 5 and 35 subjects. Oxygen was most frequently delivered by means of a non-rebreather mask for 5–60 min. Other modalities included a regular face mask, mouth piece, or head tent. In three studies, the modality for oxygen delivery was not mentioned. Arterial oxygen tensions were available for 50% of the datasets (18/36). Oxygen supplementation led to average P_a_O_2_ of 269–617 mmHg in healthy volunteers, 234–604 mmHg in CAD patients, 312–326 mmHg in patients with HF, 390–450 mmHg in patients after CABG surgery, and 350–416 mmHg in patients with sepsis. In the studies with the critically ill, all patients were intubated. In healthy volunteers, hemodynamic measurements were performed with invasive techniques (thermo- and dye dilution) up until 1972. Afterwards, noninvasive techniques such as ultrasound and bio-impedance were used. In studies with patients, all systemic hemodynamic measurements were performed invasively, except for one [19] which used bio-impedance.

**Table 1:** Study overview for healthy volunteers

Study	Year	Age (years)	Subjects (*n*/*n* male)	Administration (modality)	Exposure (min)	Intervention* (baseline/O_2_)	Measurement (method)	HD	DO_2_
Barrat-Boyes [53]	1958	28	20/NR	Mouth piece	19–60	0.21/1.0	Fick (blood)	✓	
Daly [20]	1962	21–32	9–15/NR	Mouth piece	10	0.21/1.0	Dye dilution	✓	
Foster [49]	1969	44	5/NR	Head tent	15	78/269	Dye dilution	✓	
Andersen [28]	1970	15–59	5–13/NR	NR	10	87/548	Dye dilution	✓	✓
Karetzky [26]	1971	21	8–14/1	Mouth piece	15	92/617	Fick (gas)	✓	✓
Kenmure [54]	1972	NR	20/20	NIV mask	45	91/537	Dye dilution	✓	
Harten [55]	2003	35	15/8	Face mask	5	0.21/1.0	Impedance	✓	
Smit [56]	2003	46	7/6	Mouth piece	10	0.21/1.0	MRI	✓	
Waring [57]	2003	28	8/5	Non-rebreather	10	0.21/1.0	Impedance	✓	
Anderson [58]	2005	37	30/18	Non-rebreather	5	0.21/1.0	Impedance	✓	
Rousseau [59]	2005	26	12/12	Non-rebreather	15	0.21/1.0	Cardiac echo	✓	
Bak [9]	2007	31	9/7	Non-rebreather	15	0.21/1.0	Cardiac echo	✓	
Ley [60]	2007	26	10/5	Non-rebreather	5	0.21/1.0	MRI	✓	
Kim [61]	2008	22–34	20/8	Face mask	30	0.21/1.0	Volume clamp	✓	
Bodetoft [27]	2011	26–65	16/8	Non-rebreather	15	88/383	MRI	✓	✓
Gole [35]	2011	32	10/10	Non-rebreather	15	0.21/1.0	Cardiac echo	✓	
Gao [62]	2012	27	8/4	Non-rebreather	10	0.21/1.0	Cardiac echo	✓	
Sinski [63]	2014	40	11/11	Face mask	20	0.21/1.0	Impedance	✓	
Fagoni [64]	2015	41	19/16	NR	10	0.21/1.0	Volume clamp	✓	

Studies are sorted based on year of publication

Age is reported as mean or range

* A value > 1.0 indicates an arterial partial pressure of oxygen (P_a_O_2_) in mmHg; values < 1 indicate the fraction of oxygen in the inhaled gas

*DO*_2_ oxygen delivery, *HD* hemodynamics, *MRI* magnetic resonance imaging, *NIV* noninvasive ventilation, *NR* not reported

**Table 2:** Study overview for patients

Study	Year	Age (years)	Subjects (*n*/*n* male)	Administration (modality)	Exposure (min)	Intervention* (baseline/O_2_)	Measurement (method)	HD	DO_2_
*Coronary artery disease*									
Thomas [21]	1965	61	6/6	Face mask	20	66/276	Dye dilution	✓	
Foster [49]	1969	NR	16/NR	Head tent	15	68/234	Dye dilution	✓	
Ganz [65]	1972	56	9/7	Non-rebreather	7	75/403	Dye dilution	✓	
Lecerof [66]	1974	46–59	8/8	NR	20	0.21/604	Dye dilution	✓	
Saadjian [50]	1999	62	20/15	Non-rebreather	30	77/355	Thermodilution	✓	
Mak [67]	2001	63	12/10	Non-rebreather	20	78/358	Thermodilution	✓	
*Heart failure*									
Daly [22]	1963	35–79	15/NR	Non-rebreather	10	0.21/1.0	Dye dilution	✓	✓
Haque [51]	1996	50	10/8	Non-rebreather	20	0.21/1.0	Thermodilution	✓	
Saadjian [50]	1999	68	35/26	Non-rebreather	30	75/326	Thermodilution	✓	
Mak [67]	2001	62	16/15	Non-rebreather	20	78/312	Thermodilution	✓	
Park [19]	2010	66	13/13	Non-rebreather	15	0.21/1.0	Impedance	✓	
*Intensive care unit - coronary artery bypass graft*									
Kuttila [32]	1990	52	8/8	Intubated	15	150/450	Thermodilution	✓	
Harten [33]	2005	64	15/11	Intubated	10	138/1.0	Dye dilution	✓	
Helmerhorst [34]	2017	63	22/17	Intubated	15	84/390	Waveform	✓	
*Intensive care unit - sepsis*									
Reinhart^‡^ [29]	1991	NR	20/NR	Intubated	30	113/402	Thermodilution	✓	✓
Reinhart [30]	1995	53	19/13	Intubated	30	106/416	Thermodilution	✓	✓
Rossi [10]	2007	52	14/7	Intubated	20	102/350	Brachial echo		✓

Studies are sorted based on year of publication and grouped by study population

Age is reported as mean or range

* A value > 1.0 indicates an arterial partial pressure of oxygen (P_a_O_2_) in mmHg; values < 1 indicate the fraction of oxygen in the inhaled gas

^‡^ 11 patients had ‘other cardiorespiratory insufficiencies’

*DO*_2_ oxygen delivery, *HD* hemodynamics, *MRI* magnetic resonance imaging, *NR* not reported, *P*_*a*_*O*_2_ partial pressure of oxygen

### Missing correlation coefficients

There were no studies that reported the correlation coefficient between measurements during normoxia and hyperoxia. However, some reported individual data from which these coefficients could be calculated. The average of the calculated coefficients was then used for the remaining studies and for the primary analysis. This resulted in correlation coefficients of 0.95, 0.93, 0.79, 0.95, 0.87, and 0.97 for HR [20–24], SV/SV index (SVI) [20–22, 24], CO/cardiac index (CI) [20–22, 24, 25], MAP [20–24], SVR/SVR index (SVRI) [20–24], and DO_2_ [22], respectively. The sensitivity analyses showed that the pooled effect size did not change significantly for any of the parameters when lower correlation coefficients (0.7 or 0.5) were used.

### Risk of bias

See Additional file 2 for an overview of the risk of bias scores. Objectives were clearly described in all studies and the study populations was adequately described in most. Sample-size calculations were not reported in the majority of studies. In studies with healthy volunteers, the magnitude of the intervention was verified by measuring the arterial oxygen tension in only 5/19 studies. Two used the transcutaneous oxygen tension as an indication for the change in P_a_O_2_ after oxygen inhalation. The majority of the studies with patients did measure P_a_O_2_ (13/17). Randomization (order of supplying air or oxygen) was applied in only a few studies. The blinding of either the assessor or the participant was not or inadequately described in most studies. Funnel plots revealed no evidence of publication bias.

### Meta-analysis

**Fig. 2:**
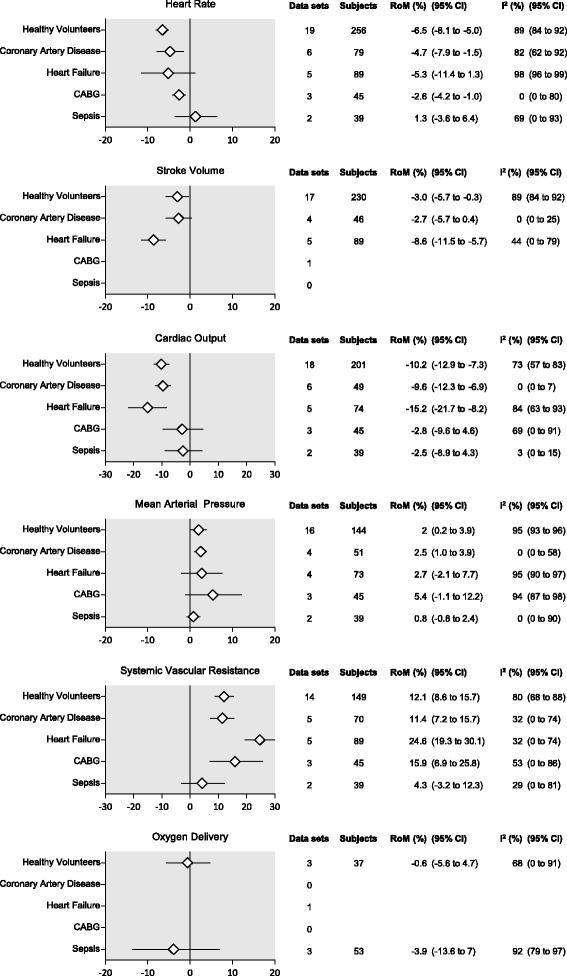
Summary effect sizes. Results of the meta-analysis of acute hyperoxia-induced changes in heart rate, stroke volume, cardiac output, mean arterial pressure, systemic vascular resistance, and oxygen delivery in healthy volunteers, and patients with coronary artery disease or heart failure, patients after coronary artery bypass graft (CABG) surgery and patients with sepsis. Summary effect sizes are expressed in percentage change from baseline (ratio of means; RoM). CI confidence interval

Figure 2 displays the summary effect sizes for heart rate, stroke volume, cardiac output, mean arterial pressure, systemic vascular resistance, and oxygen delivery for studies with healthy volunteers, and CAD, heart failure, post-CABG, and septic patients. Additional file 4 shows the forest plots with the individual studies for each subject group.

Oxygen inhalation caused a reduction in heart rate between 6.5 and 2.6%. These changes were seen in healthy volunteers (−6.5%, 95% CI −8.1% to −5.0%, *n* = 19 datasets), CAD patients (−4.7%, 95% CI −7.9% to −1.5%, *n* = 6), and CABG patients (−2.6%, 95% CI −4.2% to −1.0%, *n* = 3). Effects of hyperoxia on heart rate in heart failure (−5.3%, 95% CI −11.4% to 1.3%, *n* = 5) and sepsis patients (1.3%, 95% CI −3.6% to 6.4%, *n* = 2) were not statistically significant.

Stroke volume was measured in most studies with healthy volunteers (*n* = 17 datasets) and those with CAD (*n* = 4) and HF patients (*n* = 5), but not sufficiently in studies with post-CABG (*n* = 1) or septic patients (*n* = 0). After oxygen supplementation, stroke volume decreased by 3% (95% CI −5.7% to −0.3%) and 8.6% (95% CI −11.5% to −5.7%) in healthy volunteers and heart failure patients, respectively. No effect on stroke volume was seen in CAD patients (−2.7%, 95% CI −5.7% to 0.4%, *n* = 4).

Oxygen supplementation reduced cardiac output in all nonhospitalized individuals. The effect ranged from −10.2% (95% CI −12.9% to −7.3%, *n* = 18 studies) in healthy volunteers to −9.6% (95% CI −12.3% to −6.9%, *n* = 6) in CAD patients and −15.2% (95% CI −21.7% to −8.2%, *n* = 5) in heart failure patients. Cardiac output did not decrease significantly in post-CABG (−2.8%, 95% CI −9.6% to 4.6%, *n* = 3) or sepsis patients (−2.5%, 95% CI −8.9% to 4.3%, *n* = 2).

Mean arterial pressure increased by 2% (95% CI 0.2% to 3.9%, *n* = 16 datasets) and 2.5% (95% CI 1.0% to 3.9%, *n* = 4) in healthy volunteers and CAD patients, respectively. No statistically significant change in MAP was seen in patients with heart failure (2.7%, 95% CI −2.1% to 7.7%, *n* = 4), post-CABG surgery (5.4%, 95% CI −1.1% to 12.2%, *n* = 3), or sepsis (0.8%, 95% CI −0.8% to 2.4%, *n* = 2).

In all patient groups, except for patients with sepsis (4.3% 95% CI −3.2% to 12.3%, *n* = 2 datasets), hyperoxia increased systemic vascular resistance. In healthy volunteers, and CAD, HF, and CABG patients, the increase was 12.1% (95% CI 8.6% to 15.7%, *n* = 14), 11.4% (95% CI 7.2% to 15.7%, *n* = 5), 24.6% (95% CI 19.3% to 30.1%, *n* = 5), and 15.9% (95% CI 6.9% to 25.8%, *n* = 3), respectively.

Oxygen delivery did not change in healthy volunteers or septic patients. This variable was not measured in CAD or CABG patients. Only one study measured the effect of hyperoxia on DO_2_ in patients with heart failure, which showed no change either [22].

### Heterogeneity

As represented by the *I*^2^ statistic and its 95% CI intervals, considerable heterogeneity was found in the results for studies with healthy volunteers for all variables, except oxygen delivery. To explore this heterogeneity, we categorized studies based on several characteristics and tested the change in heterogeneity of cardiac output measurements. These categories were exposure time (≤ 0 min, 11–20 min, and > 20 min), participant blinding (yes, no/unclear), assessor blinding (yes, no/unclear), and invasiveness of the measurements performed (invasive, noninvasive). Categorization based on the magnitude of the intervention (e.g., P_a_O_2_) was not possible because only five studies reported these values. The modality used to administer oxygen was not considered as an alternative because there is known high variability in the actual F_I_O_2_ being breathed by the subject, despite using similar masks. Grouping studies with an exposure time ≤ 10 min resulted in an *I*^2^ of 0% (95% CI 0% to 6%, *n* = 9). However, in the two remaining exposure time groups (11–20, *n* = 7, and > 20 min, *n* = 3) heterogeneity remained high (78% and 79%, respectively). For the other characteristics, categorization did not substantially reduce heterogeneity.

As an alternative, we analyzed studies which appeared to deviate substantially from the summary effect estimate in either effect direction or size on probable methodological explanations (for an overview see Additional file 5). Large differences in effect size were mostly associated with ultrasound measurements. In one case almost all subjects were female, and in another the subjects were competitive divers. Although the effect of gender on the hyperoxic response is unknown, divers may have adjusted to hyperoxic oxygen tensions as they are usually exposed to hyperoxia during dives due to the increased pressure under water.

For studies in patients with CAD, heterogeneity was confined to heart rate results (*I*^2^ 82%, 95% CI 62% to 92%). In these patients, P_a_O_2_ interacted significantly with the decrease in heart rate (β = −0.03%; *p* = 0.0009), meaning that the magnitude of hyperoxia is an important modifier of the effect size. Studies in patients with heart failure showed heterogeneity in results related to changes in heart rate (*I*^2^ 98%, 95% CI 96% to 99%), cardiac output (*I*^2^ 84%, 95% CI 63% to 93%), and mean arterial pressure (*I*^2^ 95%, 95% CI 90% to 97%). Hyperoxia had variable effects in studies with patients after CABG surgery, as indicated by an *I*^2^ of 94% (95% CI 87% to 98%). Similarly, high heterogeneity existed between studies in patients with sepsis investigating the effect of oxygen supplementation on oxygen delivery (*I*^2^ 92%, 95% CI 79% to 97%). Unfortunately, due to the limited number of studies performed in these patient groups, further exploration of this heterogeneity was not possible.

## Discussion

In this systematic review and meta-analysis, we found that hyperoxia does not increase systemic oxygen delivery in healthy volunteers, heart failure patients, or septic patients. Hyperoxia reduces cardiac output and increases systemic vascular resistance, and slightly increases mean arterial pressure in healthy volunteers or nonhospitalized cardiovascular-compromised patients. In patients with sepsis, hyperoxia does not seem to effect central hemodynamics.

Oxygen supplementation is generally initiated with the intent to increase oxygen availability to cells. However, the present meta-analysis shows that “the more, the better” does not apply to oxygen. In five of the six studies in healthy volunteers [26–28], HF patients [22], and septic patients [29, 30], hyperoxia did not increase systemic oxygen delivery and, in one study with septic patients, hyperoxia even decreased oxygen delivery [10]. Although hypoxic patients benefit from a higher fraction of inspired oxygen, supplementation above normoxia seems to be futile as the hemodynamic response to hyperoxia (decrease in CO, increase in SVR) outbalances the benefit of additionally dissolved oxygen in the blood.

Oxygen inhalation reduced cardiac output by approximately 10% and increased systemic vascular resistance by 11–12% in both healthy volunteers and CAD patients. In these groups, the reduced cardiac output is predominantly driven by a reduction in heart rate rather than stroke volume. In heart failure patients, however, cardiac output decreased by 15% through a reduction in stroke volume instead of heart rate. No change in MAP was found, but SVR increased by 25%. This larger increase could be related to a combination of the increased neurohormonal activity and endothelial dysfunction seen in these patients [31]. In post-CABG surgery patients, there also was a significant increase in SVR, but without the decrease in cardiac output seen in other patients; this resulted in a tendency towards a small increase in MAP. A clear difference between hospitalized and nonhospitalized patients included in this meta-analysis is that the former may have received inotropic and vasoactive support during the study periods (e.g., dopamine and norepinephrine). It is, however, unlikely that these affected the results because drug infusion rates were kept constant during the study periods [29, 32–34]. In addition, pharmacological blockade of α- and β-receptors does not alter the hemodynamic response to hyperoxia in healthy volunteers and hyperoxia does not affect plasma (nor)epinephrine levels [35, 36]. Similarly, in isolated arteries, hyperoxia has no effect on α-receptor-mediated constriction [37]. Altogether, heart failure patients seem to be the most sensitive to the negative hemodynamic effects of hyperoxia.

Increases in SVR indicate significant arterial vasoconstriction. In humans, oxygen has been shown to induce vasoconstriction in the coronary [38, 39], brachial [40], retinal [41, 42], and cerebral vascular bed. Recent studies of the sublingual microcirculation show that hyperoxia increases heterogeneity of the microcirculation when healthy volunteers [43], CABG patients [34], or a mixed group of ICU patients [44] breathe pure oxygen, with a decrease in perfused vessel density of 15–30%. This parameter reflects the number of vessels that contribute to the exchange of oxygen and nutrients in the microcirculation. These alterations may compromise oxygen delivery on a cellular level, especially when organs are already defunct of proper perfusion due to pre-existing (vascular) pathology. This has been shown in an animal model of coronary stenosis, in which hyperoxic vasoconstriction exacerbated cardiac ischemia [45]. Supplying oxygen to patients with acute myocardial infarction increases infarct size, although it is unclear whether this is primarily due to impaired perfusion, increased generation of reactive oxygen species, or both [4, 46]. It is important to note that increases in SVR give no indication of the location of vasoconstriction. Some vascular beds may show more constriction than others. For instance, in dogs, hyperoxia increases blood flow to the kidney, liver, and intestines but reduces flow to the myocardium, pancreas, and skeletal muscle [47]. A similar redistribution was seen in pigs with fecal peritonitis and in rats with hemorrhagic shock [7, 8].

The absence of an effect of hyperoxia on SVR in septic patients is consistent with the clinical observations of vasoplegia that may occur in these patients. Although only two small studies were performed in this patient population, the lack of an effect on SVR and MAP in these patients questions the postulated beneficial effect of increasing blood pressure without the use of fluid resuscitation or vasopressors. Indeed, a randomized controlled trial which investigated this potential positive effect for patients with septic shock found no change in vasopressor requirements when patients were ventilated with pure oxygen during the first 24 h of admission [48]. This observation is in line with the result of our meta-analysis.

For most groups and parameters, considerable heterogeneity existed. We believe this is primarily caused by methodological differences. Despite using pure oxygen, the actual administered fraction of oxygen through a mask may vary substantially because of mixing with air in the absence of a perfect seal. Even when using the same mask and ventilation system, intra-individual variation in the resulting arterial oxygen tension can exist, while the hemodynamic response to oxygen seems to be P_a_O_2_-dependent [9, 27, 49–51]. Because arterial oxygen tensions were not measured in most studies, it was impossible to account for the most obvious and important possible source of heterogeneity. For the patient groups, the number of studies was insufficient to properly investigate heterogeneity. Differences in effect size may also be caused by using measuring methods that are sensitive to small changes in setup or that require additional manual processing, especially when the study is performed in an unblinded fashion. For instance, the studies in healthy volunteers with the largest decrease in stroke volume were performed with handheld ultrasound probes, a technique which is known to be highly sensitive to slight changes in measuring angles. Similarly, a large hyperoxia-induced decrease in systemic oxygen delivery in septic patients was only observed when measuring brachial blood flow with ultrasound. The unblinded study design, in combination with a measuring modality with low reproducibility, may have led to an overestimation of the true effect. On the other hand, the correlation between brachial flow and cardiac index is low, so a regional difference is not excluded. However, for the majority of studies, we do not think the methodological issues (e.g., lack of blinding or randomization) were particularly impactful because of the pre-post-test design and the use of objective endpoints [52].

## Conclusion

The present meta-analysis evaluating pre-post studies shows that there is no evidence supporting the belief that oxygen supplementation in the absence of hypoxemia increases systemic oxygen delivery. Combined with potentially significant decreases in cardiac output and increases in systemic vascular resistance in cardiac-compromised patients, we discourage superfluous oxygen supplementation.

## Additional file

Additional file 1: Search strategy. The search strategies used to search the PubMed and EMBASE strategies for eligible studies. (DOCX 15 kb)

Additional file 2: Risk of bias tool and analysis. Modified risk of bias tool used to assess the risk of bias in the included studies, along with the results of the risk of bias analysis. (DOCX 109 kb)

Additional file 3: Adjusted formulae. Formulae used in this meta-analysis, adjusted to include pre-post correlations. (DOCX 20 kb)

Additional file 4: Forest plots per group. Forest plots of the individual studies. (PDF 115 kb)

Additional file 5: Studies that deviate from the mean. Characteristics of the studies with healthy volunteers which show results that deviate substantially from the mean effect size. (DOCX 27 kb)

## Abbreviations

CABG: Coronary artery bypass graft
CAD: Coronary artery disease
CI: Confidence interval
CO: Cardiac output
DO_2_: Oxygen delivery
F_I_O_2_: Fraction of inspired oxygen
HF: Heart failure
HR: Heart rate
ICU: Intensive care unit
MAP: Mean arterial pressure
P_a_O_2_: Arterial partial pressure of oxygen
RoM: Ratio of means
SD: Standard deviation
SEM: Standard error of the mean
SV: Stroke volume
SVR: Systemic vascular resistance
