# Supplementary material for: Hemodynamic effects of acute hyperoxia: systematic review and meta-analysis
Source: Crit Care. 2018 Feb 25;22:45. doi: 10.1186/s13054-018-1968-2 (PMC6389225; doi:10.1186/s13054-018-1968-2)
Supplement: Supplementary file 1 — Search strategy. The search strategies used to search the PubMed and EMBASE strategies for eligible studies. (DOCX 15 kb) [file 13054_2018_1968_MOESM1_ESM.docx]

**Supplemental File 1** – Search strategy

| **PubMed query** |
| --- |
|  |
| Hemodynamics |
| "hemodynamics"[Mesh] OR “vascular resistance” [Mesh] OR “vascular capacitance” [Mesh] OR “vascular resistance”[tiab] OR “peripheral resistance”[tiab] OR "heart rate"[tiab] OR "cardiac output"[tiab] OR "cardiac index"[tiab] OR "stroke volume"[tiab] OR "stroke index"[tiab] OR "blood pressure"[tiab] OR "arterial pressure"[tiab] OR "hemodynamic"[tiab] OR "haemodynamic"[tiab] OR "hemodynamics"[tiab] OR "haemodynamics"[tiab] OR "microcirculation"[tiab] OR "microvascular"[tiab] OR "microcirculatory"[tiab] OR "blood flow"[tiab] |
|  |
| Hyperoxia |
| "Hyperoxia"[Mesh] OR “Oxygen/pharmacology"[Mesh] OR “hyperoxia"[tiab] OR “hyperoxaemia"[tiab] OR “hyperoxic"[tiab] OR “oxygen supplementation"[tiab] OR “oxygen administration"[tiab] OR “oxygen induced"[tiab] OR “oxygen-induced"[tiab] OR “oxygen exposure"[tiab] OR “oxygen inhalation"[tiab] OR “oxygen insufflation”[tiab] OR “oxygen breathing”[tiab] OR “100% Oxygen"[tiab] OR “pure oxygen"[tiab] OR “O2 supplementation"[tiab] OR “O2 administration"[tiab] OR “O2 induced"[tiab] OR “O2 exposure"[tiab] OR “O2 inhalation"[tiab] OR “O2 insufflation”[tiab] OR “100% O2"[tiab] OR“pure O2"[tiab] |
|  |
| Humans |
| "Humans"[Mesh] OR "volunteers"[MeSH] OR "Research Subjects"[MeSH] OR "volunteers"[MeSH] OR "Patients"[MeSH] OR "Human experimentation"[MeSH] OR "healthy subjects"[tiab] OR "humans"[tiab] OR "patients"[tiab] OR "volunteers"[tiab] OR "men"[tiab] OR "man"[tiab] OR "woman"[tiab] OR "women"[tiab] OR "person"[tiab] OR "persons"[tiab] |
|  |
| **EMBASE query** |
|  |
| Hemodynamics |
| ‘hemodynamics’/exp OR ‘vascular resistance’/exp OR ‘vascular resistance’:ti,ab OR ‘peripheral resistance’:ti,ab OR ‘heart rate’:ti,ab OR ‘cardiac output’:ti,ab OR ‘cardiac index’:ti,ab OR ‘stroke volume’:ti,ab OR ‘stroke index’:ti,ab OR ‘blood pressure’:ti,ab OR ‘arterial pressure’:ti,ab OR ‘hemodynamic’:ti,ab OR ‘haemodynamic’:ti,ab OR ‘hemodynamics’:ti,ab OR ‘haemodynamics’:ti,ab OR ‘microcirculation’:ti,ab OR ‘microvascular’:ti,ab OR ‘microcirculatory’:ti,ab OR ‘blood flow’:ti,ab |
|  |
| Hyperoxia |
| ‘Hyperoxia’/exp OR ‘hyperoxia’:ti,ab OR ‘hyperoxaemia’:ti,ab OR ‘hyperoxic’:ti,ab OR ‘oxygen supplementation’:ti,ab OR ‘oxygen administration’:ti,ab OR ‘oxygen induced’:ti,ab OR ‘oxygen-induced’:ti,ab OR ‘oxygen exposure’:ti,ab OR ‘oxygen inhalation’:ti,ab OR ‘oxygen insufflation’:ti,ab OR ‘oxygen breathing’:ti,ab OR ‘100% Oxygen’:ti,ab OR ‘pure oxygen’:ti,ab OR ‘O2 supplementation’:ti,ab OR ‘O2 administration’:ti,ab OR ‘O2 induced’:ti,ab OR‘O2 exposure’:ti,ab OR ‘O2 inhalation’:ti,ab OR ‘O2 insufflation’:ti,ab OR ‘100% O2’:ti,ab OR ‘pure O2’:ti,ab |
|  |
| Humans |
| ‘human’/exp OR ‘research subject’/exp OR ‘patient’/exp OR ‘human experiments’/exp OR ‘male’/de OR ‘female’/de OR ‘healthy subjects’:ti,ab OR ‘humans’:ti,ab OR ‘patients’:ti,ab OR ‘volunteers’:ti,ab OR ‘men’:ti,ab OR ‘man’:ti,ab OR ‘woman’:ti,ab OR ‘women’:ti,ab OR ‘person’:ti,ab OR ‘persons’:ti,ab |
