# Supplementary material for: Hemodynamic effects of acute hyperoxia: systematic review and meta-analysis
Source: Crit Care. 2018 Feb 25;22:45. doi: 10.1186/s13054-018-1968-2 (PMC6389225; doi:10.1186/s13054-018-1968-2)

**Supplemental file 2 –** Risk of bias tool and analysis

The table below is based on the Risk of Bias tool by the American National Institutes of Health

**Possible answers:** Yes (Low risk of bias) / No (High risk of bias) / Unclear (Unclear risk of bias) / Not applicable

| **Component** | **Comments/Clarification** |
| --- | --- |
| Was there a clear study objective? | Did the authors describe their goal in conducting this research? Is it easy to understand what they were looking to find? |
| Was the study population well described? | Did the authors describe relevant characteristics of the subjects investigated? |
| Was there a sample size calculation? | Was the study size based on a power calculation? Did the authors otherwise explain why they included a certain number of participants/patients? |
| Was the intervention clearly described/delivered? | Was the method of oxygen administration clearly described and did the authors validate the administration in any way? |
| Was the intervention applied randomly? | Did the participants receive oxygen or air in a randomized fashion? |
| Was the study population stable? | For healthy volunteers; did the authors mention a period of rest or accustomation before commencing the measurements?  For patients; did the authors describe hemodynamic stability of the patient prior to/during the study period? |
| Were carry-over effects investigated? | If the intervention was applied randomly, was the residual effect of oxygen investigated or commented on? If oxygen was always administered second, then this component is not applicable |
| Were outcome measures consistently assessed? | Were the measurements described in the methods section measured and reported in all participants? Or was the occurrence of missing values reported and explained? |
| Were participants blinded to the intervention? | Was blinding of the participants completely described and was the method adequate? Sedated patients were considered ‘blinded’ |
| Were the assessors blinded to the intervention? | Was blinding of the assessor completely described and was the method adequate? |
| Were appropriate statistical tests performed? | Were proper tests performed and reported? This also includes adequate reporting of measures of variability. |

**Risk of Bias analysis**


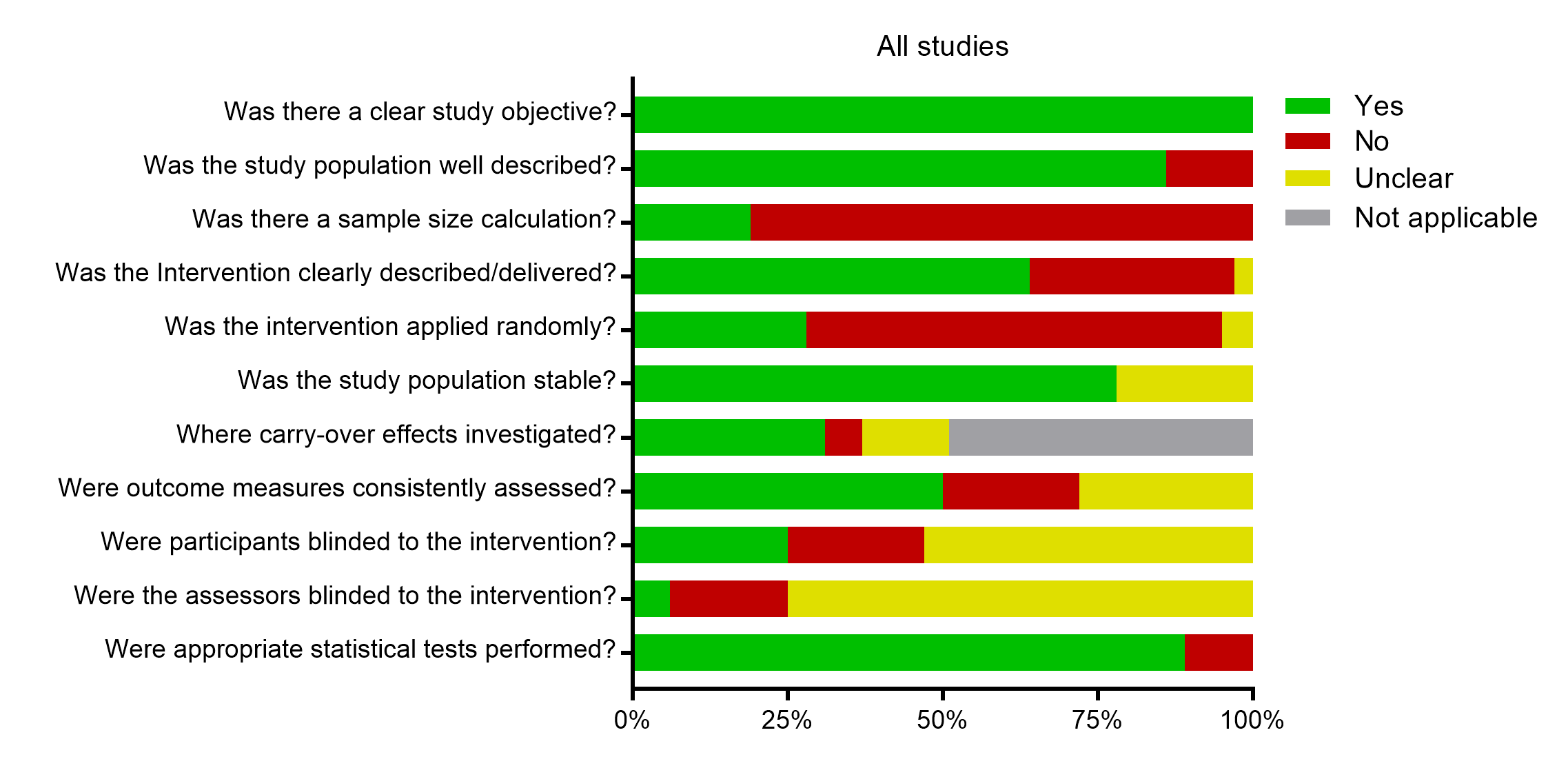

Supplement: Supplementary file 2 — Risk of bias tool and analysis. Modified risk of bias tool used to assess the risk of bias in the included studies, along with the results of the risk of bias analysis. (DOCX 109 kb) [file 13054_2018_1968_MOESM2_ESM.docx]
