# Supplementary material for: Hemodynamic effects of acute hyperoxia: systematic review and meta-analysis
Source: Crit Care. 2018 Feb 25;22:45. doi: 10.1186/s13054-018-1968-2 (PMC6389225; doi:10.1186/s13054-018-1968-2)
Supplement: Supplementary file 3 — Adjusted formulae. Formulae used in this meta-analysis, adjusted to include pre-post correlations. (DOCX 20 kb) [file 13054_2018_1968_MOESM3_ESM.docx]

**Supplemental File 3** – Formulas used for the Ratio of Means random effects model

Original formula as published by Friedrich et al. [1]

$$\ln\left( RoM \right)=\ln(\frac{{mean}_{exp}}{{mean}_{contr}})$$

$$SE[\ln\left( RoM \right)= \sqrt{\frac{1}{n_{exp}}\left( {\frac{{SD}_{exp}}{mean}}_{exp} \right)^{2}+\frac{1}{n_{contr}}\left( {\frac{{SD}_{contr}}{mean}}_{contr} \right)^{2}}$$

$$RoM=exp\{\left[ \ln\left( RoM \right) \right]\}$$

$$95\% CI=exp\{[\ln(RoM)] \pm1.96\times SE[ln(RoM)]\}$$

Adjusted formula including pre-post correlation and transformation of Ratio of means to % change:

$$\ln\left( RoM \right)=\ln(\frac{{mean}_{oxygen}}{{mean}_{air}})$$

$$SE[\ln\left( RoM \right)= \sqrt{\left( \frac{1}{n}\left( {\frac{{SD}_{ox}}{mean}}_{ox} \right)^{2}+\frac{1}{n}\left( {\frac{{SD}_{air}}{mean}}_{air} \right)^{2} \right)-\left( 2*r*\left( {\frac{{SD}_{air}}{{mean}_{air}}}/\sqrt{n} \right) *\left( {\frac{{SD}_{ox}}{{mean}_{ox}}}/\sqrt{n} \right) \right)}$$

$$RoM=(exp\left\{ \left[ \ln\left( RoM \right) \right] \right\}-1)*100$$

$$95\% CI=((exp\{\left[ \ln(RoM)] \pm1.96\times SE[ln(RoM)]\} \right)-1)*100$$

**References**

1. Friedrich JO, Adhikari NKJ, Beyene J. Ratio of means for analyzing continuous outcomes in meta-analysis performed as well as mean difference methods. J Clin Epidemiol. 2011;64: 556–564. doi:10.1016/j.jclinepi.2010.09.016
