# Supplementary material for: Hemodynamic effects of acute hyperoxia: systematic review and meta-analysis
Source: Crit Care. 2018 Feb 25;22:45. doi: 10.1186/s13054-018-1968-2 (PMC6389225; doi:10.1186/s13054-018-1968-2)
Supplement: Supplementary file 4 — Forest plots per group. Forest plots of the individual studies. (PDF 115 kb) [file 13054_2018_1968_MOESM4_ESM.pdf]

Healthy volunteers

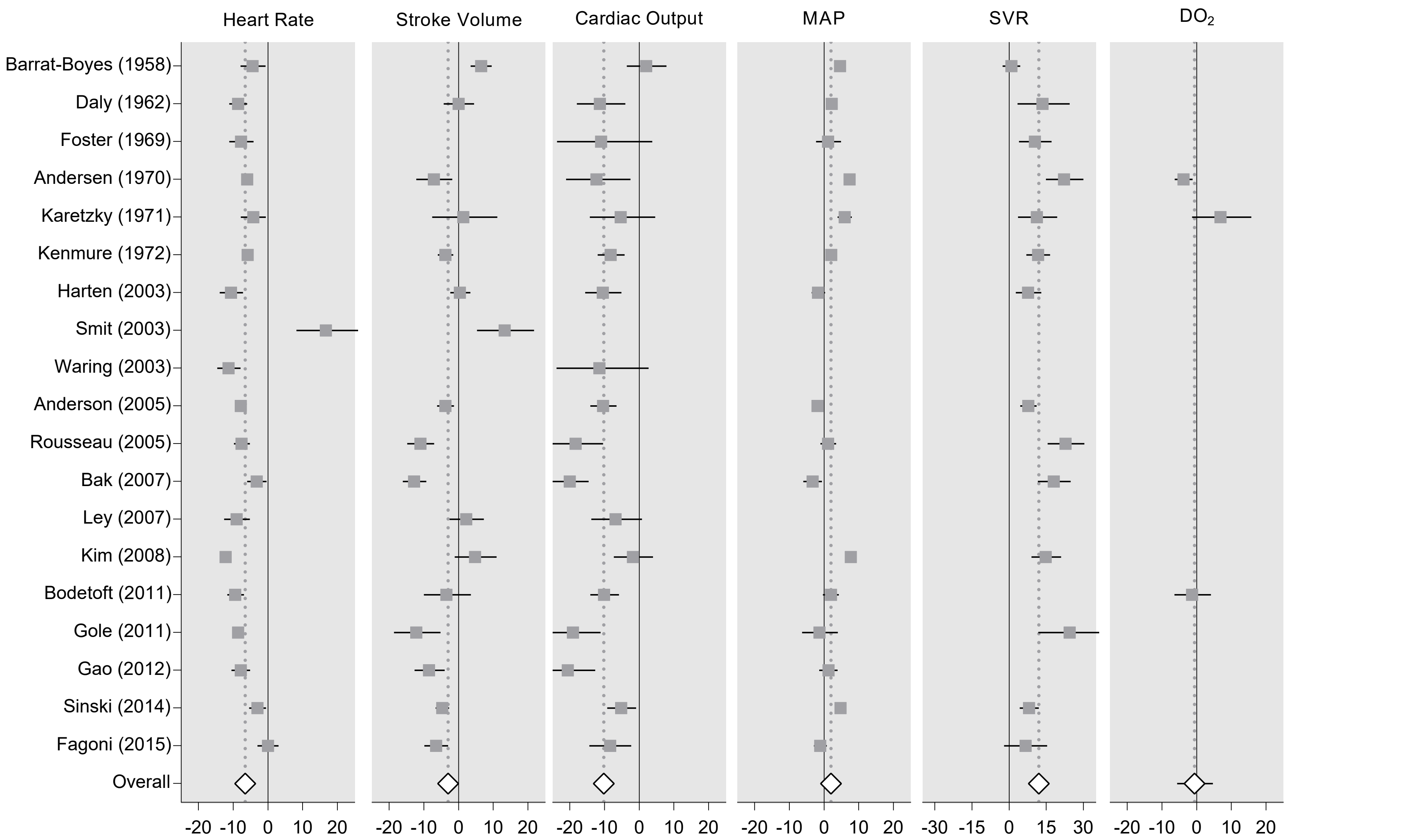

	Heart Rate	Stroke Volume	Cardiac Output	MAP	SVR	DO_2
**RoM (%)**	-6.5 (-8.1 to 5.0)	-3 (-5.7 to -0.3)	-10.2 (-12.9 to -7.3)	2 (0.2 to 3.9)	12.1 (8.6 to 15.7)	-0.6 (-5.6 to 4.7)
**I^2 (%)**	89 (84 to 92)	89 (84 to 92)	73 (57 to 83)	95 (93 to 96)	80 (68 to 88)	68 (0 to 91)

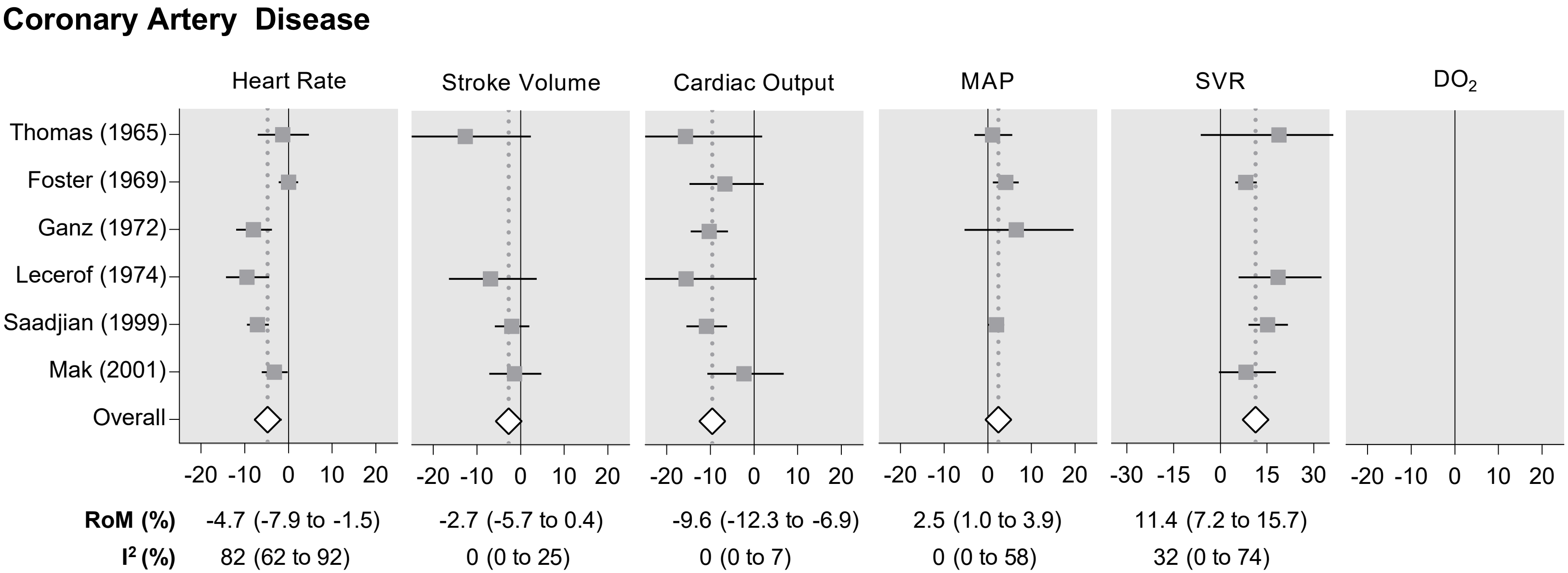
Coronary Artery Disease
Heart Rate
Stroke Volume
Cardiac Output
MAP
SVR
DO$_2$
Thomas (1965)
Foster (1969)
Ganz (1972)
Lecerof (1974)
Saadjian (1999)
Mak (2001)
Overall
-20 -10 0 10 20
-30 -15 0 15 30
RoM (%)
-4.7 (-7.9 to -1.5)
-2.7 (-5.7 to 0.4)
-9.6 (-12.3 to -6.9)
2.5 (1.0 to 3.9)
11.4 (7.2 to 15.7)
I^2 (%)
82 (62 to 92)
0 (0 to 25)
0 (0 to 7)
0 (0 to 58)
32 (0 to 74)

Heart Failure

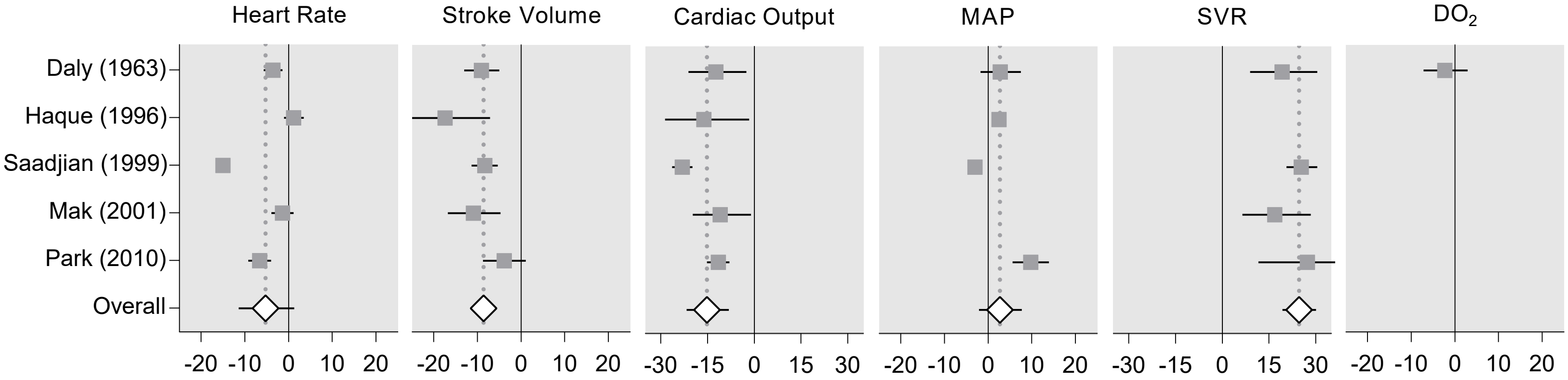

	Heart Rate	Stroke Volume	Cardiac Output	MAP	SVR	DO_2
**RoM (%)**	-5.3 (-11.4 to 1.3)	-8.6 (-11.5 to -5.7)	-15.2 (-21.7 to -8.2)	2.7 (-2.1 to 7.7)	24.6 (19.3 to 30.1)	
**I^2 (%)**	98 (96 to 99)	44 (0 to 79)	84 (63 to 93)	95 (90 to 97)	32 (0 to 74)	

Coronary Artery Bypass Grafting

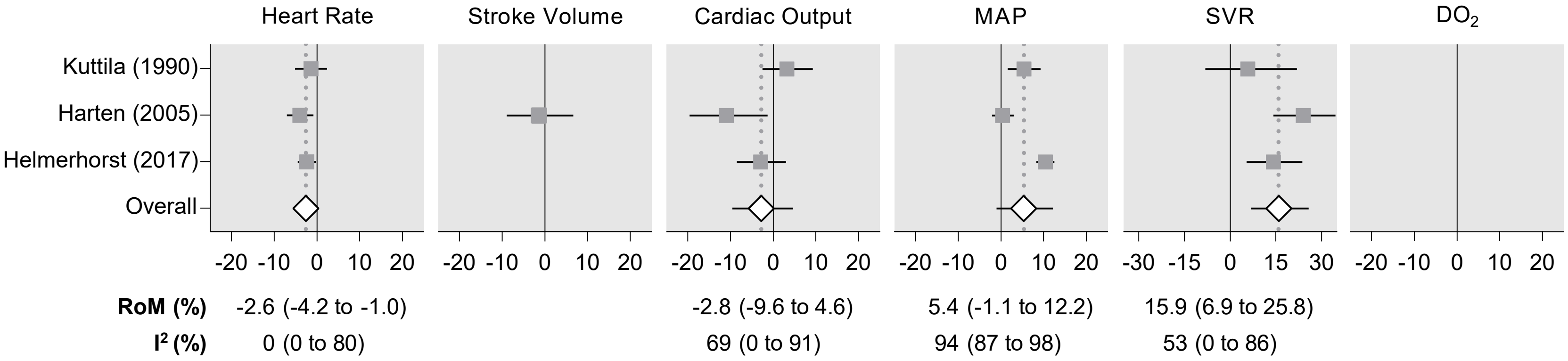

Sepsis

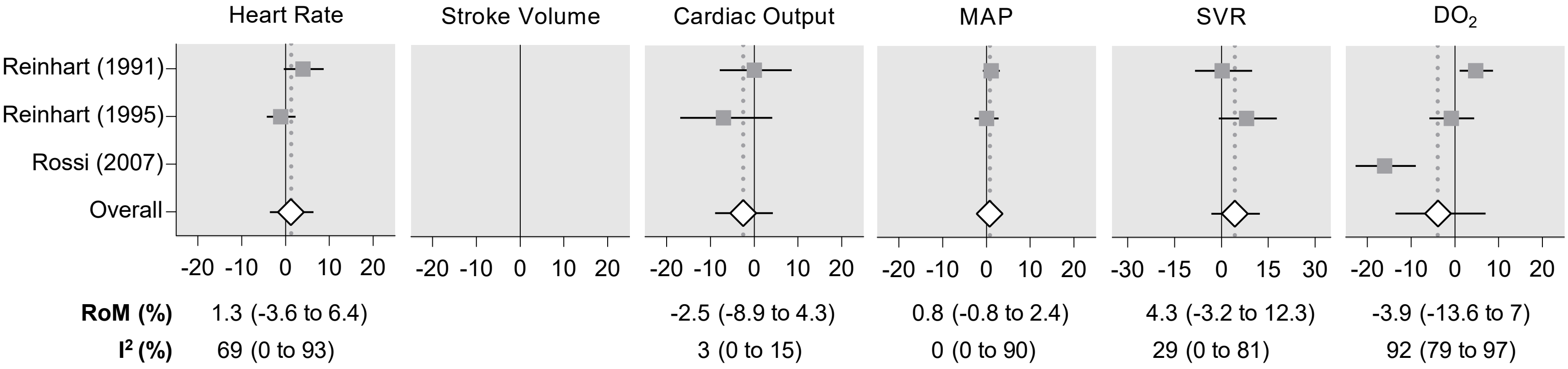
Heart Rate
Stroke Volume
Cardiac Output
MAP
SVR
DO_2
Reinhart (1991)
Reinhart (1995)
Rossi (2007)
Overall
-20 -10 0 10 20
-30 -15 0 15 30
RoM (%)
1.3 (-3.6 to 6.4)
-2.5 (-8.9 to 4.3)
0.8 (-0.8 to 2.4)
4.3 (-3.2 to 12.3)
-3.9 (-13.6 to 7)
I^2 (%)
69 (0 to 93)
3 (0 to 15)
0 (0 to 90)
29 (0 to 81)
92 (79 to 97)
