# Supplementary material for: Hemodynamic effects of acute hyperoxia: systematic review and meta-analysis
Source: Crit Care. 2018 Feb 25;22:45. doi: 10.1186/s13054-018-1968-2 (PMC6389225; doi:10.1186/s13054-018-1968-2)
Supplement: Supplementary file 5 — Studies that deviate from the mean. Characteristics of the studies with healthy volunteers which show results that deviate substantially from the mean effect size. (DOCX 27 kb) [file 13054_2018_1968_MOESM5_ESM.docx]

**Supplemental file 5 –** Studies that deviate from the mean

| **1^st^ Author** | **Observed effect** | | | | | | **Possible explanation** | |
| --- | --- | --- | --- | --- | --- | --- | --- | --- |
|  | HR | SV | CO | MAP | SVR |  | |  |
| Overall | ↓↓ | ↓ | ↓↓ | ↑ | ↑↑ | Not applicable | |  |
| Barrat-Boyes [1] |  | ↑ |  | ↑↑ |  | Stroke volume was calculated from cardiac output based on the arterial-venous oxygen content difference. MAP measured by hand on paper recordings. | |  |
| Andersen [2] |  |  |  | ↑↑ |  | MAP was measured intra-arterially or by cuff. Unclear when which method was used. | |  |
| Karetzky [3] |  |  |  | ↑↑ |  | Participants were mostly females (11/14). MAP measured on paper recordings. | |  |
| Smit [4] | ↑ | ↑ |  |  |  | MRI measurements were traced by hand. Blinding unclear. | |  |
| Rousseau [5] |  | ↓↓ | ↓↓↓ |  |  | Measurements were made with ultrasound. Assessor blinding unclear. | |  |
| Bak [6] |  | ↓↓ | ↓↓↓ |  |  | Measurements were made with ultrasound. Assessor blinding questionable. | |  |
| Kim [7] | ↓↓↓ |  |  | ↑↑ |  | No obvious methodological explanation. | |  |
| Gole [8] |  | ↓↓ | ↓↓↓ |  |  | Measurements were made with ultrasound. Method of blinding not described. | |  |
| Gao [9] |  | ↓↓ | ↓↓↓ |  |  | Measurements were made with ultrasound. Assessor blinding unclear. | |  |
| Fagoni [10] | *↔* |  |  |  | *↔* | Subjects were competitive divers. | |  |
| Abbreviations: HR, heart rate; SV, Stroke Volume; CO, Cardiac Output; MAP, Mean Arterial Pressure; SVR, Systemic Vascular Resistance. Clarification of symbols: ↑/↓ Small increase/decrease; ↑↑/↓↓, moderate increase/decrease; ↓↓↓, strong decrease; *↔,* no effect*.* | | | | | | | |  |

**References**

1. Barratt-Boyes BG, Wood EH. Cardiac output and related measurements and pressure values in the right heart and associated vessels, together with an analysis of the hemo-dynamic response to the inhalation of high oxygen mixtures in healthy subjects. J Lab Clin Med. 1958;72–90.

2. Andersen A, Hillestad L. Hemodynamic responses to oxygen breathing and the effect of pharmacological blockade. Acta Med Scand. 1970;188:419–24.

3. Karetzky MS, Keighley JF, Mithoefer JC. The effect of oxygen administration on gas exchange and cardiopulmonary function in normal subjects. Respir Physiol. 1971;12:361–370.

4. Smit HJ, Vonk-Noordegraaf A, Marcus JT, Van Der Weijden S, Postmus PE, De Vries PMJM, et al. Pulmonary vascular responses to hypoxia and hyperoxia in healthy volunteers and COPD patients measured by electrical impedance tomography. Chest. 2003;123:1803–1809.

5. Rousseau a., Bak Z, Janerot-Sjöberg B, Sjöberg F, Janerot-Sjoberg B, Sjoberg F. Acute hyperoxaemia-induced effects on regional blood flow, oxygen consumption and central circulation in man. Acta Physiol Scand. 2005;183(3 PG-231-240):231–240.

6. Bak Z, Sjoberg F, Rousseau A, Steinvall I, Janerot-Sjoberg B. Human cardiovascular dose-response to supplemental oxygen. Acta Physiol. 2007;191(1 PG-15-24):15–24.

7. Kim YK, Jun IG, Kim SR, Hwang JH, Cho SK, Han SM, et al. Using 100% oxygen does not alter the cardiovascular autonomic regulation during non-invasively simulated haemorrhage in healthy volunteers. J Int Med Res. 2008;227–236.

8. Gole Y, Gargne O, Coulange M, Steinberg JG, Bouhaddi M, Jammes Y, et al. Hyperoxia-induced alterations in cardiovascular function and autonomic control during return to normoxic breathing. Eur J Appl Physiol. 2011;111:937–946.

9. Gao Z, Spilk S, Momen A, Muller MD, Leuenberger UA, Sinoway LI. Vitamin C prevents hyperoxia-mediated coronary vasoconstriction and impairment of myocardial function in healthy subjects. Eur J Appl Physiol. 2012;112(2 PG-483-492):483–492.

10. Fagoni N, Sivieri A, Antonutto G, Moia C, Taboni A, Bringard A, et al. Cardiovascular responses to dry resting apnoeas in elite divers while breathing pure oxygen. Respir Physiol Neurobiol. 2015;219:1–8.
